# Burnout among surgeons before and during the SARS-CoV-2 pandemic: an international survey

**DOI:** 10.1186/s40359-023-01517-4

**Published:** 2024-01-25

**Authors:** Mostafa Shalaby, Ahmed M. ElSheikh, Hosam Hamed, SURG-SAT-19 Collaborative Group, Ahmed Elsheik, Ahmad Sakr, Amgad Fouad, Amr Kassem, Hossam Elfeki, Khaled Madbouly, Khalid H. Alzahrani, Khalid Marzouk, Mahmoud Ali, Mohamed Alaa Abdelmoez Helal, Mohamed Elsorogy, Mohamed Farid, Nicola Di Lorenzo, Pierpaolo Sileri, Steven Wexner, Wael Khafagy, Ademola Adeyeye, Alaa El-Hussuna, Alice Frontali, Avanish Saklani, Benedettao Lelpo, Daniela Molena, Diwakar Pandey, Elena Karbovnichaya, Francesco Pata, Gabrielle H. Van Ramshor, Gaetano Gallo, Gaya Spolverato, Gianluca Pellino, Giulia Bagaglini, Ines Rubio-Perez, Ionut Negoi, Isabella Frigerio, Jovan Juloski, Marijana Ninkovic, Marzia Franceschilli, Mina Azer, Sergey Efetov, Simona Ippoliti, Zoe Garoufalia, Mohammad Rafi Fazli, Agron Dogjani, Harieche Abdennour Abderahim Cherfa, Tilioua Omar, Javier Minoldo, José Maria Alvarez Gallesio, Matias Quesada, Annica Bacher, Stephan Kropshofer, Florian Ponholzer, Philip Tesik, Philipp Gehwolf, Sevim Isci, Stefan Uranitsch, Valeria Berchtold, Elgun Samadov, Abdulmenem Abualsel, Ashrarur Rahman Mitul, S. M. Nazmul Islam, Aude Vanlander, Charles Van Praet, Elke Van Daele, Hanne Vanommeslaeghe, Jasper Stijns, Kessewa Abosi-Appeadu, Martijn Depuydt, Mathias Allaeys, Van Nieuwenhove Yves, Ramiro Colleoni, Mihail Slavchev, Aly Elbahrawy, Jessica G. Y. Luc, Karen Milford, Ivan Romic, Alessio Monti, Ashraf Haydal, Mads Falk Klein, Miranda E. K. Ocklind, Sabah Anwar Hadi, Abdallah Alqasaby, Abdelazim Elganash, Adel Goda Hussein Daibes, Adham Elsaied, Ahmad Elhattab, Ahmad Lotfy, Ahmed Alnashar, Ahmed Abd Elbaset Elsayed Abu Elnour, Ahmed Abdelhalim, Ahmed Abdelhamid, Ahmed Abdellatif, Ahmed Abdelmohsen, Ahmed Abdelrafee, Ahmed Adel Elhawary, Ahmed Azmy Zidan, Ahmed Eleshra, Ahmed Elkafoury, Ahmed Ezz, Ahmed Ezzat Elghrieb Abdelmomen, Ahmed Farag Elkased, Ahmed Fawzy, Ahmed G. Elkhouly, Ahmed Gamal Abouelfetouh Ibrahim Hemidan, Ahmed Hosam Eldin Hasan Abbas, Ahmed Mahmoud Ahmed Ismail, Ahmed Mohamed Attia, Ahmed Mohammed Farid, Ahmed Mostafa Elnakash, Ahmed Negida, Ahmed Soliman, Ahmed Taki-Eldin, Ali Almahdy Ali Albadry, Aly Sanad, Amira Alsayed Abdelhai Elbatal, Amr Elgazar, Amr Saleh, Andrew Fahiem, Anwar Yahya A. Mohamed, Ashraf Nageeb, Ashraf S. Elmetwally, Ayman Alkhalegy, Ayman El-Wakeel, Ayman Shemes, Bashir A. Fadel, Basma Waseem Lutfi, Doaa Ali, Khaled Samir Abolnasr, Ehab Gamal, Emad Abdallah, Emad Ali Ahmed, Eman Abdalla Mohamed Salem, Esmael Ali Hamed, Essam Elshikh, Farazdaq Enad, Fetoh Alaaeldin Fetoh Sarhan, Galal Abouelnagah, Gamal Hassan El Tagg, Gehad Atef, George Samir Habib Shaker, Hatem Beshir, Hazem M. Zakaria, Hesham Barbary, Hesham Elgendy, Hesham Sharaf, Hisham Elnaghi, Hosam Elghadban, Ibrahim Elzayat, Ibrahim Fakhr, Ibrahim Sallam, Ibrahim Tharwat Mohamed Abdelmoneim, Islam Elnemr, Karem Shahin Mohamed Zewar, Khaled Elalfy, Khaled Sabet, Khaled Yousery Ibrahim Mansour, Khalid Abdalla Abdelgadir Osman, Maher Elesawi Kamel Elgaly, Maher Shams, Mahmoud Abozeid, Mahmoud M. Mohammed, Mahmoud Mohamed Elkatt, Mahmoud Yahia Samaha, Marolla Maher Eskander Mikhael, Medhat M. H. A. Khalil, Moaaz Alhendawey, Mohamad Elrefai, Mohamed A. Gabr, Mohamed Abdelaziz Mohamed Abdalla Mohamed Ali Fayed, Mohamed Abdelmaksoud, Mohamed Abouelmagd Salem, Mohamed Adel Mohamed Mohamed, Mohamed Adel Nabeeh, Mohamed Ahmed Abdelhalim Ahmed Elsayed, Mohamed Ahmed Abdelmonem, Mohamed Anwar Abdel Razik Ali, Mohamed Eldemery, Mohamed Elmesery, Mohamed Fikry, Mohamed Gharbia, Mohamed I. Omar, Mohamed Ibrahim Elmoghazy, Mohamed Jomma Ghazala, Mohamed Korayem Fattouh Hamed, Mohamed Metwally, Mohamed Mohamed Hamdy Arnouse, Mohamed Mohsen Amen, Mohamed Mokhtar Amary, Mohamed Mosaad Kandel, Mohamed Mostafa Abuzeid, Mohamed Rabea, Mohamed Ramadan Sobh, Mohamed Taman, Mohammad Fathy, Mohammad Montaser Hassan Moustafa, Mohammad Zuhdy, Mohammed Adel, Mohammed Alaa, Mohammed Alawady, Mohammed El Edassy, Mohammed Mustafa Hassan Mohammed, Mohammed Nabil Eldesouki, Mohammed Said Mahmoud Salim, Mohammed Sanad, Mohsen George Khalaf, Mohsen Michael Henes, Momen Abdelglil, Mona Mhmoud Mohmmed, Morsi Mohamed Morsi Abdelkhalik, Mosab Shetiwy, Mostafa Elshazli, Mostafa Hegazy, Mostafa Mahmoud Ahmed, Mostafa Mohammed Abdelhalim, Mostafa Shahein, Mostafa Sofan, Muhammed Alaa Moukhtar Hammad, Mustafa Ahmad, Nader Milad, Nehal Farouk, Omnia Eldesouky, Omnia Y. Mohamed, Osama Abdel Salam Mahadel, Osama Gaarour, Radwan Abdelsabour Torky, Raheem El-Gohary Abd Elhafez, Ramy Magdy Adly, Ramy Mikhael Nageeb, Salah Hamdi, Sameh Gamal, Sameh Hany Emile, Samer Regal, Sayed Abdelrasheed, Shady Ahmed Elzeftawy, Sohib Mohammed Mohammed Khashshan, Tamer Ashraf, Tamer Khafagy, Tamer Nabil, Tarek Abdelazim, Tarek Taher Rizk, Wesam Amr, Yousef Mohamed Yousef, Youssef Abdel Aziz Youssef, Antonio Castaldi, Antonio Fiore, Ariola Hasani, Aurora Mariani, Claire Dagorno, D’Alessandro Antonio, Giuliano Izzo, Giulio Addari, Giuseppe Mangiameli, Lo Dico Rea, Luca Pio, Marco Paci, Police Andrea, GSerena De Fatico, Tartaglia Elvira, Alejandro Daniel Lira Schuldes, Eslam Rihan, Gabriela Moeslein, Hans Lederhuber, Ibram Botros, Ismail Jaman, Johannes Doerner, John Rezk Hanna Elseberbihy, Kareem El Sherbiny, Mostafa Ghonim, Amir Mikrish, Mina Aziz, Mohamed Hatm, Rami Archid, Samuel Elkess Morcos Gendy, Sufian Ahmad, Alexandros Charalabopoulos, Anastasia Prodromidou, Argyrios Ioannidis, Eustratia Mpaili, Garyfallia Boukorou, Georgios Papadopoulos, Theodore Liakakos, Vasileiadou Styliani, Abhishek Agrawal, Amita Jain, Arshad Rashid, Asif Mehraj, Swagata Brahmachari, Harish Neelamraju Lakshmi, Kushagra Vishwakarma, Lalit Parida, Meenakshi Sharma, Mohammad Zaieem, Murtaza Makasarwala, Rigved Nittala, Sanjeev Kumar, Sharma Vikrantmr, Sheikh Junaid, Somyaa Khuller, Vinal More, Abeer Abdul Hameed Ahmed, Adil Alomieri, Arkan Shubber Alhamdany, Muslim Ka Del, Ghadah Najm, Nawras Falah Lateef, Deborah Mcnamara, Mohammed Elkassaby Abdelmageed, Mudassar Majeed, Albert Troci, Alberto Porcu, Alessandra Marano, Alessandro Di Bartolomeo, Alessandro Giani, Alessandro Giardino, Alfonso Canfora, Andrea Balla, Andrea Barberis, Andrea Belli, Andrea Borasi, Andrea Manetti, Andrea Mingoli, Andrea Morini, Angela Maurizi, Angelo Alessandro Marra, Angelo Gabriele Epifani, Angelo Iossa, Angelo Parello, Anna Guida, Anna Maffioli, Anthony Kevin Scafa, Antonino Spinelli, Antonio Matarangolo, Arcangelo Picciariello, Brunella Pirozzi, Bruno Cirillo, Carlo Gazia, Carlo Ratto, Caterina Foppa, Chiara Marafante, Chierici Andrea, Cinzia Tanda, Claudio Guerci, Cristine Don, Daniele Zigiotto, Denise Coniglio, Diego Sasia, Diego Visconti, Donato F. Altomare, Eleonora Guaitoli, Emanuele Botteri, Enrico Pinotti, Fabio Martinelli, Fabio Uggeri, Fabrizio Bàmbina, Federica Falaschi, Federico Costanzo, Filippo La Torre, Flavio Milana, Francesca Abbatini, Francesca De Lucia, Francesca Paola Tropeano, Francesco Colombo, Francesco Ferrara, Francesco Litta, Francesco Maria Carrano, Francesco Orlando, Francesco Roscio, Francesco Selvaggi, Gabriella Giarratano, Gianluca Pagano, Giorgio Lisi, Giulio Argenio, Giuseppa Zancana, Giuseppe Cavallaro, Giuseppe Frazzetta, Grasso Mariateresa, Guido Sciaudone, Ivan Vella, Leandro Siragusa, Letizia Santurro, Lorenzo Ferri, Lorenzo Petagna, Luca Ferrario, Ludovica Pitoni, Marcello Filograna Pignatelli, Marco Angrisani, Marco Giugliano, Marco Inama, Marco V. Marino, Marco Veltri, Maria Carmela Giuffrida, Maria Paola Menna, Marina Valente, Matteo Rottoli, Matteo Sacchi, Matteo Uccelli, Maurizio Rho, Mauro Garino, Mauro Montuori, Michela Campanelli, Monica Zese, Nadia De Falco, Nicola Cillara, Nicolò Maria Mariani, Nicolò Tamini, Ottavio Adorisio, Paola Campennì, Paolina Venturelli, Paolo Bernante, Paolo Sapienza, Pasquale Cianci, Patrizia Marsanic, Pierfrancesco Lapolla, Piero Tecchio, Pietro Familiari, Pietro Fransvea, Placido Bruzzaniti, Redan Hassan, Riccardo Pirovano, Roberto Rimonda, Salomone Di Saverio, Sara Di Carlo, Teresa Perra, Tommaso Campagnaro, Valentina Testa, Valeria Andriola, Virgilio Michael Ambrosi Grappelli, Vita Capizzi, Vito Chiarella, Vittoria Bellato, Katsuhiko Yanaga, Mohamed Farouk, Ahmad Uraiqat, Mahmoud Almasri, Ambrose Nabwana, Mark M. W. Siboe, Njoroge P. W, Githu Njoroge, Jh. Ilkul, Ralph Ombati Obure, Yusuf Palkhi, Ali Alkhayat, Ali Sayed Ali, Amgad Nashaat Abdel Malek, Emad Fahim Abdelsayed, Tarek Zahra, Larissa Ayoub, Fadi Sleilati, Rany Aoun, Nassib Algatanesh, Nura Ahmed Fieturi, Jen Siang Ng, Andrés Vega Díaz, Erik Efrain Sosa Duran, José Eaazim Flores Guerrero, Manuel Meza Jasso, Manuel SSalas Flores, Marcos José Serrato Felix, Victor Manuel Pinto Angulo, Abdelhadi Mejdane, Abdelmounaim Aitali, Benzakour Amal, Aziz Zentar, Ahmed Bensaad, El Alami Yacir, Fassi Fihri Mohamed Jawad, Mohamed Ghassane Rachid, Mohamed Maliki-Alaoui, Mouaqit Ouadii, Ouazni Mohammed, Nyan Thein, Dinesh Prasad Koirala, Denise Hilling, Sjaak Pouwels, Abiodun Idowu Okunlola, Adeyinka Adejumo, Akinola Akinmade, Asimiyu Adekunle Shittu, Ayodele Samuel Oluyomi, Azeez Lateef Abiodun, Bashir Lawal, Clement Odion, Ademola Popoola, Edward Jolayemi, El-Zaki Shomoye, Funmilola Olanike Wuraola, Grace Eke, Henry Abiyere, Ige Oluwasuyi, Ihediwa George, Iloba Gabriel Njokanma, Isiaka Aremu, Julius Kolajo Dare, Lukman Abdur-Rahman, Misbahu Haruna Ahmad, Mobolaji Adewale Oludara, Mohammad Aminu Mohammad, Ojajuni Adeoluwa, Oladele Situ, Peter Agbonrofo, Raji Taofiq Kewulere, Yakubu Aliyu, Yusuf Adebowale, Ahmed Galala, Satish Rao, Aasma Waleed, Aatif Inam, Abdul Razaque Shaikh, Ahmad Uzair Qureshi, Aneeqah Din Muhammad, Arooj Ahmed, Asad Ali Kerawala, Mohammad Aslam, Asma Mehr, Ayesha Javed, Farooq Ahmad, Haroon Javaid Majid, Hassan Ahmed, Irfan Daudi, Khalid Akhtar, Khurram Niaz, Mariyah Anwer, Mohammed Amir, Muhammad Amir Hanif, Muhammad Asif, Muhammad Asif Raza, Muhammad Imran Khokhar, Muhammad Khurram Jameel, Muhammad Nasir, Muhammad Salman Shafique, Mujammad Ateeb, Munawar Nadeem, Rahmat Ullah Shah, Shahzad Hussain Waqar, Shahzad Alam Shah, Talat Waseem, Tariq Ghafoor, Tauseef Fatima, Umar Bashir, Erick Ivan Huaman Gonzales, Luis Angel Garcia Ruiz, Carla Freitas, Xavier De Sousa, Ahmed Al-Bahrani, Carlos Antonio Sanchez Portela, Elsayed Aly Elgazar, Eloy Morasen Robles, Irfan Jan Khan, Lutfi Jarboa, Mahwish Khawar, Miguel Jose Pinto Echevarria, Moataz M. Bashah, Salahaldeen Dawdi, Shameel Musthafa, Syed Muhammad Ali, Cezar Ciubotaru, Eduard-Alexandru Bonci, Mihai-Stefan Muresan, Stoica Bogdan, Tanase Ioan, Albina Zubayraeva, Aleksandr Derinov, Alexander Zakharenko, Anastasia Novikova, Andrey Bashlachev, Ayrat Kaldarov, Berelavichus Stanislav, David Gorin, Dmitriy Puzenko, Ekaterina Kazachenko, Erkin Ashimov, Iuliia Medkova, Ivan Ignatov, Kochetkov Viktor Sergeevich, Lyudmila Sidorova, Michail Kiselev, Michail Danilov, Ogoreltsev Aleksandr, Sergey Rodimov, Tatiana Garmanovs, Yury Kitsenko, Nekoval Valery, Ntezamizero Japhet, Abdulrahman Sibiany, Abdelhalim Saadeldin, Abdelrahman Abuosba, Abdulbari Mohammed Alawadhi, Abdulhamid Alharbi, Abdullah Althumali, Abdullah Alghuliga, Abdullah Alotaibi, Abdullah Fayez Abduraboh, Abdullah Kateb, Abdullah Sindy, Abdulmohsen Al Eisa, Abdulrahman Alotaibi, Abdulrhman Almulhim, Adel Ali Aljawhari, Ahmad Mahmoud Abozeid, Ahmad Saad, Ahmed Alqarni, Ahmed Alwan, Ahmed Alwusaibie, Ahmed Bafaraj, Ahmed Eldeeb, Ahmed Tarabay, Mahfoudh Mohammed, Alhanouf Alhedaithy, Alhassan Hesham Almaghrabi, Ali Ibrahim Eldawy Abed, Alqahtani Ali Abdullah, Anmar Semilan, Mohamed Farag, Essa Khudhayr, Marwah Hussain, Ghanem Abbas, Heba Alqudaihi, Abdulrahman Alotaibi, Yousra Abualnaja, Abelnasser Shaheen, Ashraf Abdelazeem Mohamed Mubarak, Bandar Idrees A. Ali, Barrag Alhazmi, Bilal Ahmed Hijazi, Chadi Abdulrahman, Charles Olajide Oyedepo, Heythem Alzamel, Elsanousi Ibrahim Sabir Tairab, Munir A. Alsuwaimel, Soha Hejazi, Emad Alnoqaidan, Fade Ahmed Alhussien, Fadi Sami Jallad, Faisal Khadwardi, Faisal Saleh Alghamdi, Feras Haddad, Fozan Sauri, Haitham Alafghani, Haitham Alfalah, Hamada Gad, Hamdy Haggag Ebrahim Aboelmagid, Hamed Ibrahim, Hany M. Elzayady, Hatem Abdelrahman Ahmed Sharafeldin, Hatem A. Sembawa, Haytham Alabbas, Hazem Abbas, Hesham Elgamal, Homoud Alawfi, Humood Al-Sadery, Hussien Ali Abdelmotaleb, Ibrahim Al Hassn, Ishag M. Mudawi, Islam Nekhala, Kareem Elsanhoury, Khalid Babieker Said, Khalid A. Albeshri, Khalid Albahooth, Khalid Fathelrahman Bakier Mohammed, Khalid Mohammad Ibrahim Asar, Luqman Osman, Mahdi Alzamanan, Mahmoud Alnabarawi, Majid Althobaiti, Mohamed Abdelmoneim Elsayed, Mohamed Al Naeb, Mohamed Salah Eldin Hassan, Mohamed Sayed Abdelhamid, Mohammad Alyami, Mohammad Amin Mirza, Mohammad Sayouh, Mohammed Amer Alkhayat, Mohammed Basendowah, Mohammed Ghunaim, Mohammed Khalid Alhussaini, Mohammed Khoj, Mohammed Sbaih, Muhammad Ahmad Saeed, Muhammad Zulfiqar Ali, Nabil Yassin Tammam Abdelaziz, Nadim Malibary, Nael Abdo, Nasser Mohammed Amer, Neamat Ahmed Ali Al Turki, Norah Durayb, Nouf Yassin, Nouf Akeel, Noureddine Larbi, Ofays Alsallum, Omar AAbu Suliman, Osama Elsherbiny, Osama Abusalem, Ibrahim Altedlawi Albalawi, Raid Abdullah Abutalib, Rayan Alarabi, Roaa Ghazi Khan, Saleh Alazzam, Saleh Alghamdi, Salem Alsawat, Sami Salim, Sarah Alshukr, Saud Alzahrani, Smain Golea, Tumadher Alowairdhi, Usama Salman, Wael Abusiam, Wael Abualkhair, Wael Saber, Wail Tashkandi, Waleed Alhazmi, Waleed Tashkandi, Wassim Abou Yassine, Yaser Ahmad Alshabi, Yaser Ibrahim, Yasser Shahin, Yassin Ibrahim, Yousef Aljathlany, Yousef Alnahas, Yousef Alrashidi, Zubair Wali, Abdourahmane Ndong, Mamadou Ba, Papa Mamadou Faye, Dragana Arbutina, Ljiljana Milic, Vladica Cuk, Abdinafic Mohamud Hussein, Jeannie Mccaul, Laurie Bertels, Linda Pohl, Marion Arnold, Nomonde Mbatani, Pj Oosthuizen, Shreya Rayamajhi, Susan Vosloo, Uzair Jooma, Aitor Landaluce-Olavarria, Alba Vázquez-Melero, Alberto Marcos, Alejandro Puerto Puerto, Alicia Ruiz De La Hermosa, Ana Senent-Boza, Bakarne Ugarte-Sierra, Beatriz Cros Montalbán, Beatriz Martin-Perez, Caroina Gonzalez Gomez, Enrique Colás-Ruiz, Esther Garcia Santos, Fatima Senra, Ismael Mora-Guzmán, Jana Dziakova, Jeancarlos J. Trujillo Díaz, Jesús Silva, Juan Luis Blas Laina, Luis Tallon-Aguilar, Marcello Di Martino, Mario Franco Chacón, Matteo Frasson, Mikel Prieto Calvo, Monica Millan, Patricia Tejedoe, Sonia Pérez-Bertólez, Víctor Turrado-Rodríguez, Abdelrhman Azhari Mohammed Elsanosi, Duaa Abdalbakheet, Mohamed Ahmed, Omer El Faroug H. Salim, Mohamed Youssef, Carlotta Barbon, Amal Bouchrika, Houcine Maghrebi, Issam Loukil, Alp Yildiz, Ayberk Dursun, Baris Gulcu, Bulent Calik, Burak Eral, Değercan Yeşilyurt, Fatih Yakar, Furkan Atakan Akin, Gizem Kilinc, Gülberk Uslu, Korhan Tuncer, Mehmet Ali Koc, Sezai Leventoğlu, Selman Sokmen, Semra Demirli Atici, Tayfun Kaya, Ümit Akın Dere, Yasemin Kırmızı, Kavuma Daniel Ssenono, Herman Lule, Ronald Mbiine, Ahmed Hamza, Shabeer Ali, Saidalavi Padinhare Peediyakkal, Gopala Pillay Varma, Haidar Aal Mussa, Hayder Makki Al-Masari, Mina Shehata, Moham Seiam, Muhammad Akram Abdul Aziz, Nessrein Nimir, Ritu Khare, Shahid Rashid, Shuiab Kazim, Zafar Gondal, Ahmed Elshawadfy Sherif, Ahmed Ghanem, Ahmed Hazem I. Helmy, Ahmed Ibrahim, Ahmed Mohammed Elshaer, Ahmed Msm Marzouk, Alessandro Paolo Tamburrini, Alessandro Parente, Alexander Light, Angela Diamantopoulou, Baljit Singh, Binay Gurung, Claire Frauenfelder, Cosimo Alex Leo, Dimitri Raptis, Dixa Thakrar, Thumuluru Kavitha Madhuri, Efthymia Tsounaki, Emanuele Garreffa, Fiammetta Soggiu, George Stavrou, Hwei Jene Ng, Hani Tabasi, Hazem Nasef, Ioannis D. Kostakis, James Jeffery, Janindra Warusavitarne, Jon Lund, Kamran Qurashi, Kapil Sahnan, Kin Seng Tong, Luca Orecchia, Mandeep Kaur, Mariam Zaidi, Mario Ganau, Mohamed Ali Gad Hassan, Nathan Curtis, Nikita Bhatt, Nikolaos Machairas, Noman Zafar, Omar Toma, Panchali Sarmah, Majid Bassuni, Justin Davies, Sami Shawer, Sherif Shawer, Sophia Lewis, Sivaraman Subramanian, Suhaib Ahmad, Uqba Nadeem, Aidan Njau, Aley Eldin Tohamy, Andrea M. Pakula, Andrea Simioni, Bennie L. Jarvis, Georgios P. Skandalakis, Hosai Todd Hesham, Isaac A. Isaiah, Jennifer Villwock, Linda W. Martin, Melissa Kress, Merry Sebelik, Sanaz Lathan, Shirin Towfigh, Stefan D. Holubar, Steve Demeester, Mohammed Mohammed Hasan Alshehari, Saif Ali Ghabisha, Shehab Ahmed Ali Abdulatef, Waheeb Al-Kubati, Yasser Abdurabo Obadiel, Alexander Gots, Mildred Nakazwe, Jackson Chipaila, Dennis Mazingi

**Affiliations:** 1 Department of General Surgery, Mansoura University, 60 ElGomhouria Street, Mansoura, Dakahliya 35516 Egypt; 2 Department of Quality and Patient Safety, Security Forces Program Hospital Makkah, Makkah, Kingdom of Saudi Arabia

**Keywords:** Survey, Burnout, Surgeon, Pandemic, SARS-COV2, COVID-19, Training, Trainee

## Abstract

**Background:**

SARS-CoV-2 pandemic has had many significant impacts within the surgical realm, and surgeons have been obligated to reconsider almost every aspect of daily clinical practice.

**Methods:**

This is a cross-sectional study reported in compliance with the CHERRIES guidelines and conducted through an online platform from June 14th to July 15th, 2020. The primary outcome was the burden of burnout during the pandemic indicated by the validated Shirom-Melamed Burnout Measure.

**Results:**

Nine hundred fifty-four surgeons completed the survey. The median length of practice was 10 years; 78.2% included were male with a median age of 37 years old, 39.5% were consultants, 68.9% were general surgeons, and 55.7% were affiliated with an academic institution. Overall, there was a significant increase in the mean burnout score during the pandemic; longer years of practice and older age were significantly associated with less burnout.

There were significant reductions in the median number of outpatient visits, operated cases, on-call hours, emergency visits, and research work, so, 48.2% of respondents felt that the training resources were insufficient. The majority (81.3%) of respondents reported that their hospitals were included in the management of COVID-19, 66.5% felt their roles had been minimized; 41% were asked to assist in non-surgical medical practices, and 37.6% of respondents were included in COVID-19 management.

**Conclusions:**

There was a significant burnout among trainees. Almost all aspects of clinical and research activities were affected with a significant reduction in the volume of research, outpatient clinic visits, surgical procedures, on-call hours, and emergency cases hindering the training.

**Trial registration:**

The study was registered on clicaltrials.gov “NCT04433286” on 16/06/2020.

**Supplementary Information:**

The online version contains supplementary material available at 10.1186/s40359-023-01517-4.

## Introduction

Burnout is a multidimensional construct. Melamed et al. [1] proposed that emotional exhaustion, physical fatigue, and cognitive weariness are the core of burnout. [2] The prevalence of burnout among physicians has recently been cited at between 30 and 65% across specialties. A recent systematic review reported a mean burnout rate of 34.6% among 20,560 surgeons [3]. The literature shows that burnout has negative consequences from personal and professional aspects, resulting in increased medical errors due to burned-out physicians [4]. The World Health Organization (WHO) included burnout in the 11th Revision of the International Classification of Diseases (ICD-11) under the chapter "*Factors influencing health status or contact with health services*" [5].

The emergence of coronavirus disease-2019 (COVID-19), caused by the novel coronavirus-2 (SARS-CoV-2), has had a tragic impact on people's lives and habits. One year later, there are a total of 117,799,584 confirmed cases of COVID-19, including 2,615,018 deaths [6]. Surgeons have been obligated to reconsider almost every aspect of daily clinical practice by cancelling elective surgeries and scaling back outpatient clinics. Trainees have been at the forefront of the pandemic, with a resultant wide range of stress and burnout [7, 8].

In this cross-sectional study we aim to highlight the burden of burnout during the pandemic compared to the pre-pandemic status.

## Methods

This study was a cross-sectional design investigating the burden of burnout among surgeons, and was conducted via an online survey reported in compliance with the Checklist for Reporting Results of Internet E-Surveys (CHERRIES) [9]. Surgeons irrespective of specialties, genders, level of experiences, countries, and hospitals' type were invited to participate through an open survey with a convenient sample including all eligible respondents.

### Survey design, development, pretesting, and distribution

The survey was proposed by a consensus of surgeons, which started with reviewing the literature for relevant data by two consultant surgeons (HH and MS), then revised by a healthcare quality consultant (AS). Subsequently, a pilot group tested the survey, including trainee and consultant surgeons of varying experience and years of practice. The survey was further refined until the final version was reached.

The survey consisted of a semi-structured English language questionnaire that included 3 sections with 17 questions in its final iteration. The first sector of the questionnaire covered participant demographics and baseline characteristics (Q1-10) and the next two sectors compared the participant experience before and during the pandemic with focus on job satisfaction (Q12), burnout (Q13), and career practice (Q14), as well as the role of the participant (Q15) and the availability of resources (Q16) during the pandemic. Finally, an open-ended general question asking what interventions would have helped you better during the pandemic (Q18) was included. Q11 asked the participant's willingness to share his/her name. All questions were Likert-type scales with the "***neutral***" response representing no change during the chronologic continuum. We employed the validated Shirom-Melamed Burnout Measure (SMBM) as an indicator for the measurement of burnout [1, 10]. A full copy of the survey is included in Supplementary materials.

The survey was conducted through an online survey development cloud-based software (SurveyMonkey®; San Mateo, CA, USA). Participants of the study were aware of the nature of the survey and informed that they could potentially be listed as co-authors, if they agreed.

Potential participants were reached through social media platforms like Twitter, LinkedIn, Facebook, and WhatsApp. The survey was sent via the Egyptian Society of Surgeons' email list, the Egyptian Society of Colon and Rectal Surgeons, the Women in Surgery Society, and the Open-Source Research Collaborative Group. Furthermore, it was distributed through WhatsApp groups of young members of both the European Society of Coloproctology (Y-ESCP) and the Italian Society of Colon and Rectal Surgeons (Y-SICCR). Responses were collected from June 14th, 2020, to July 15th, 2020. Respondents were not able to review or change their answers once submitted.

### IRB approval, clinical trial registration, and consenting

This study has been approved by the local Institutional Review Board (IRB) at Mansoura Faculty of Medicine, Egypt in concordance with the Helsinki Declaration Principals, then registered with a unique identifier (NCT04433286 on 16/06/2020) at the ClinicalTrials.gov. An introductory statement regarding the study's purpose, number of questions, and the time required to complete the survey was available. The potential respondents were voluntaries to complete the survey and all confidential personal information was optional. A signed consent was not required; however, informed consent was obtained virtually from all respondents when they chose to complete the survey which was sufficient for the purpose of the study. No prior registration or login was required to complete the survey.

The study's primary outcome was the burden of burnout during the pandemic compared to the pre-pandemic status. Secondary outcomes highlighted the different aspects of burnout, the effect of different participant demographics and characteristics on burnout, and the effect of participant role and career practice during the pandemic on burnout.

### Data handling, data protection, and statistical analysis

Data were extracted from the SurveyMonkey® (SurveyMonkey Inc., San Mateo, California, USA; Main Website: www.surveymonkey.com) into Microsoft Excel® sheet (Microsoft Corp, Redmond, Washington, USA). Only one of the study's principal investigators (AS) had full access to the collected data. Furthermore, all confidential data were de-identified. The collected data were coded, processed, and analyzed using SPSS™ version 23 (IBM, Armonk, USA). Variables were expressed using mean ± standard deviation (SD), or median and normal range, and percentage. Data were tested for normal distribution using the Shapiro Wilk test. Quantitative data, if normally distributed, were expressed as mean ± SD with the paired samples t-test was used to assess the difference between two dependent groups. Whereas, if non-normally distributed quantitative data were expressed as median and range with the Wilcoxon-signed rank test was used to assess the difference between two dependent groups.

For qualitative data, the Chi-Square test was used to compare two or more groups; in case of more than 25% of cells must count less than 5 in tables (> 2*2), the Monte Carlo test was run as a correction for the Chi-Square test. The Marginal Homogeneity test was used to assess the difference between two dependent groups of categorical variables in more than two classes.

Correlation analysis was evaluated with Spearman’s correlation to test the correlation between two variables with non-parametric quantitative data. The value of the test expressed as (r), and the values are interpreted as follows; a positive value indicates a direct correlation, and negative correlation indicates an inverse correlation, while (r) from (0: 0.3) or (0: -0.3) indicates a weak correlation, (r) from (0.3: 0.6) or (-0.3: -0.6) indicates a moderate correlation, and (r) from (0.6: 1) or (-0.6: -1) indicates strong correlation. Significant test results are quoted as two-tailed probabilities. The significance level was tested for all tests mentioned above, expressed as the probability of (*P*-value), and considered significant if 0.05 or less.

## Results

**Fig. 1:**
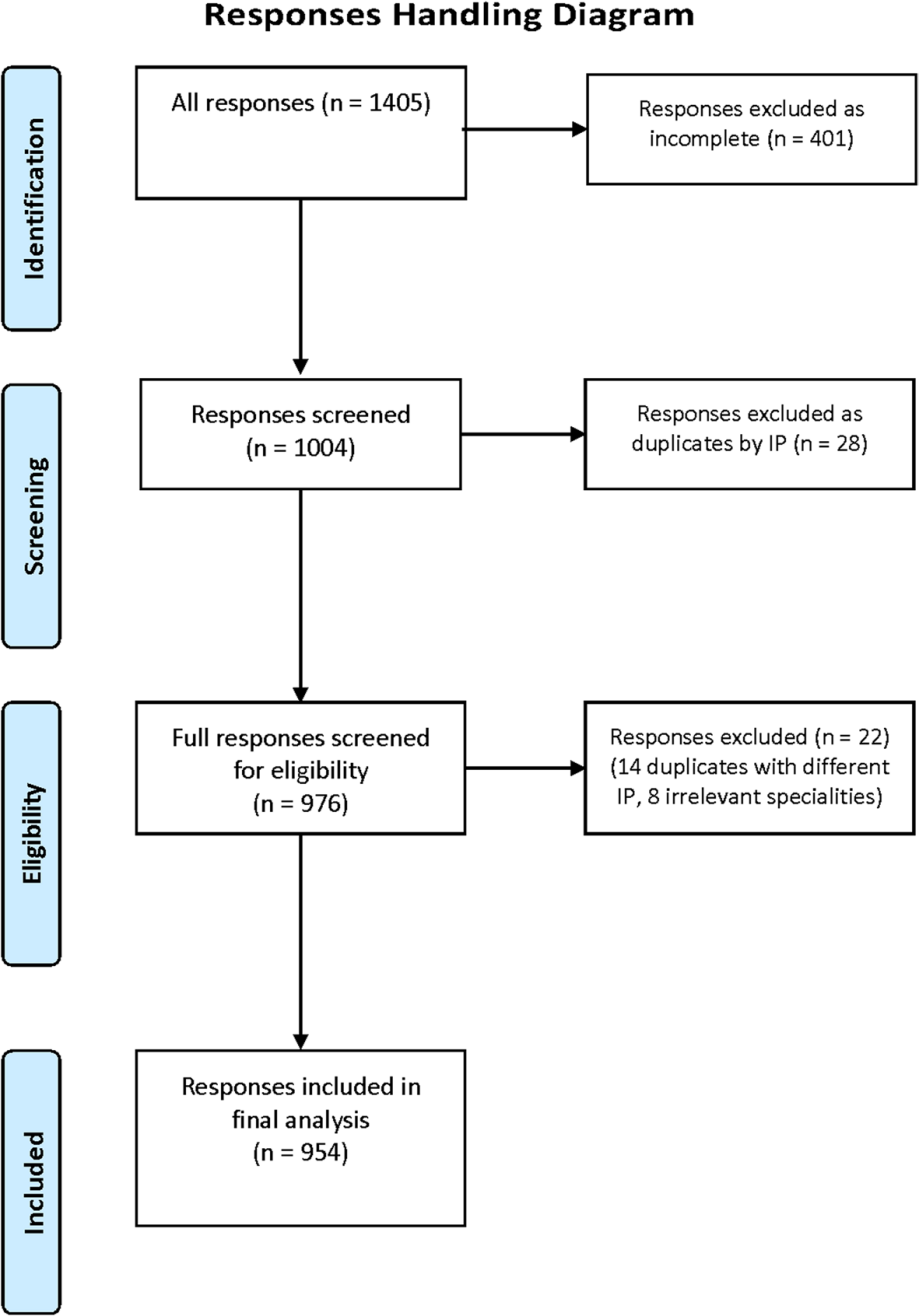
The process of responses' handling

There were a total of 1405 respondents during the collection period; 401 responses were deemed incomplete and excluded. In addition, 42 duplicates identified by IP addresses and 8 respondents were from irrelevant specialties and were also excluded, leaving a total of 954 valid responses for a completion rate of 67.9%. Eighty-three (8.7%) respondents preferred to remain anonymous. Figure 1 shows the process of responses' handling.

### Respondents' demographics and basal characteristics

**Fig. 2:**
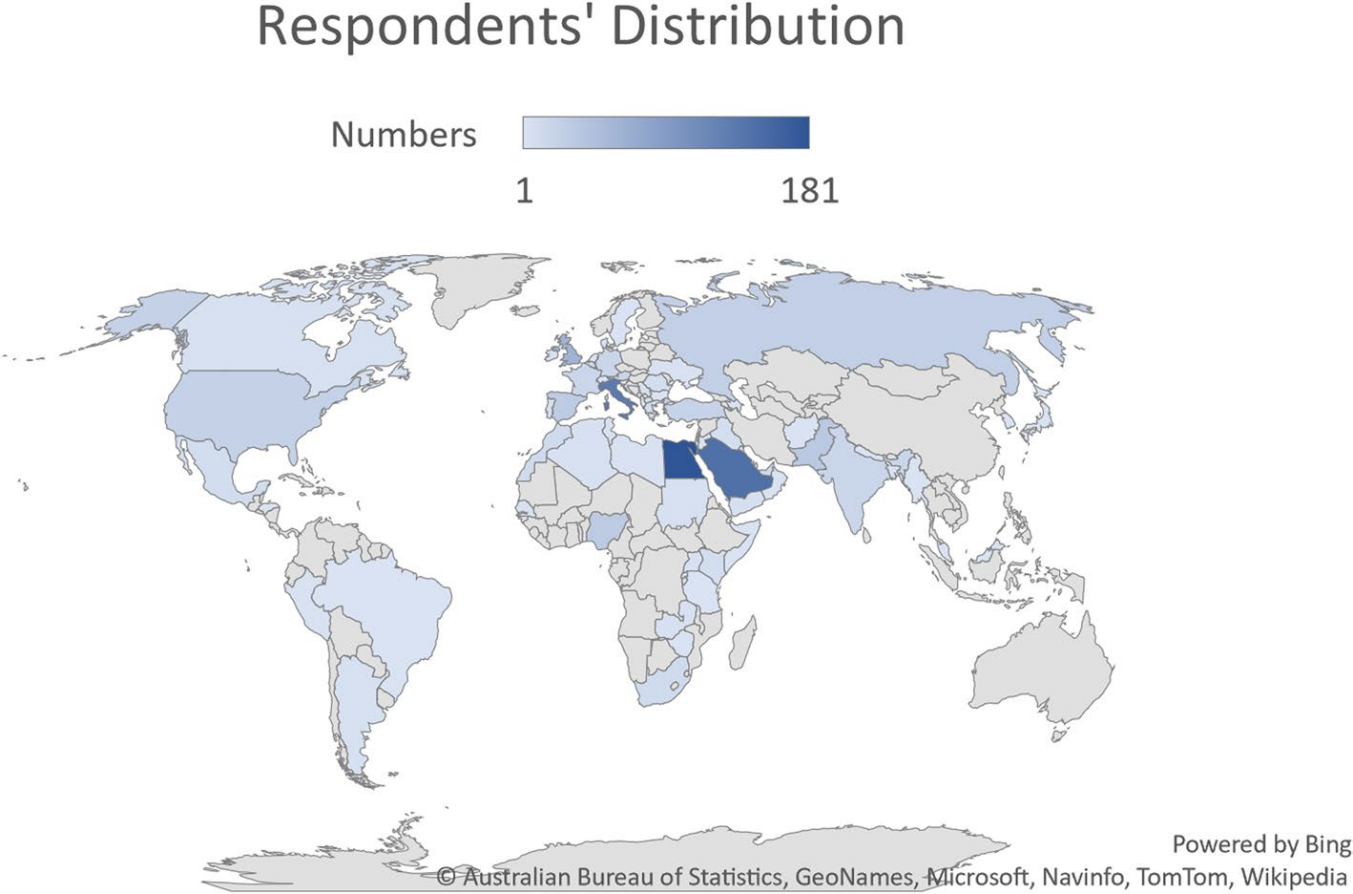
The distribution of respondents per countries

Respondents identified as male in 78.2%, female in 21.5%, and 0.3% preferred not to state their gender; the median age was 37 (range; 23 and 77) years. Respondents were from high-income, middle-income, and low-income countries in 56.6%, 41.9% and 16%, respectively [11]. They were married, single, divorced, and widowed in 74.2%, 22.3%, 2.7%, and 0.7%, respectively; 63.2% had children while 36.8% did not. Figure 2 shows the distribution of respondents per country.

Regarding practice, 39.5% were consultants, 37.1% were specialists, and 23.4% were trainees. Overall, the median years of practice was 10 (range; 0–45) years. The majority (*n* = 657; 68.9%) of respondents were general surgeons and 55.7% of all respondents were affiliated with academic institutions compared with 17.2% to non-academic institutions; 6.5% were in private practice and 20.6% were in mixed practices. Furthermore, 95.4% of respondents indicated that they work in urban communities compared to 4.6% in rural areas. More detailed characteristics of respondents are shown in Table 1.

**Table 1:** Q1-10 Participant demographics and baseline characteristics

	**Participant No (%)**
Total	954
In what country do you work?^a^	
High-income	539 (56.5%)
Upper middle-income	95 (10%)
Lower middle-income	304 (31.9%)
Low-income	16 (1.7%)
What is your age? (in years)	
Mean ± SD	39.28 ± 9.84 years
Median (range)	37 (23–77) years
What is your gender?	
Male	746 (78.2%)
Female	205 (21.5%)
Prefer not to mention	3 (0.3%)
Which of the following best describes your current relationship status?	
Married	708 (74.2%)
Divorced	26 (2.7%)
Widowed	7 (0.7%)
Single	213 (22.3%)
Do you have any children?	
Yes, all 18 or over	89 (9.3%)
Yes, one or more under 18	514 (53.9%)
No	351 (36.8%)
How many years do you have in surgical practice?	
Mean ± SD	12.49 ± 9.28 years
Median (range)	10 (0–45) years
What is your current designation?	
Resident/ Trainee	223 (23.4%)
Specialist/ Senior Specialist	354 (37.1%)
Consultant	377 (39.5%)
What is your specialty?	
General Surgery	657 (68.9%)
Orthopedic Surgery	36 (3.8%)
Pediatric Surgery	34 (3.6%)
Obstetrics and Gynecology	15 (1.6%)
Urology Surgery	23 (2.4%)
Vascular Surgery	21 (2.2%)
Otolaryngology	26 (2.7%)
Cardiothoracic Surgery	26 (2.7%)
Neurosurgery	25 (2.6%)
Plastic Surgery	15 (1.6%)
Others	76 (0.8%)
In what type of community do you work?	
City or urban community	910 (95.4%)
Rural community	44 (4.6%)
In what type of institution do you work?	
Academic	531 (55.7%)
Non-academic	164 (17.2%)
Private	62 (6.5%)
Mixed	197 (20.6%)

^a^Country classification based on World Bank rank [1]

### Burnout before and during the COVID-19 pandemic

Overall, there was a significant increase in the mean burnout score during the pandemic from 18.05 ± 5.4 to 19.33 ± 6.51 (*p* < 0.001). The only question with no significant difference was "*I feel tired*", with the majority of respondents choosing "*sometimes*" before (37.9%) and during (32%) the pandemic (Table 2). For the remaining questions showed significant difference between the pre-pandemic status and during the pandemic. The Spearman's rank-order correlation showed only age and years of practice showed a significant negative correlation with burnout, both before and during the pandemic.

**Table 2:** Q 13. Manifestations of burnout

	**Before COVID 19 Pandemic; N (%)**	**During COVID 19 Pandemic; N (%)**	*P* value
I feel tired			
Never	33 (3.5%)	64 (6.7%)	0.181
Infrequent	110 (11.5%)	162 (17.0%)	
Sometimes	362 (37.9%)	305 (32%)	
Frequent	359 (37.6%)	287 (30.1%)	
Always	90 (9.4%)	136 (14.3%)	
I have no energy for going to work in the morning			
Never	165 (17.3%)	165 (17.3%)	< 0.001
Infrequent	291 (30.5%)	237 (24.8%)	
Sometimes	332 (34.8%)	246 (25.8%)	
Frequent	123 (12.9%)	217 (22.7%)	
Always	43 (4.5%)	89 (9.3%)	
I feel fed up			
Never	156 (16.4%)	154 (16.1%)	< 0.001
Infrequent	255 (26.7%)	186 (19.5%)	
Sometimes	341 (35.7%)	295 (30.9%)	
Frequent	164 (17.2%)	230 (24.1%)	
Always	38 (4.0%)	89 (9.3%)	
I feel like my “batteries” are “dead”			
Never	206 (21.6%)	193 (20.2%)	< 0.001
Infrequent	275 (28.8%)	244 (25.6%)	
Sometimes	299 (31.3%)	246 (25.8%)	
Frequent	134 (14.0%)	201 (21.1%)	
Always	40 (4.2%)	70 (7.3%)	
I feel burned out			
Never	218 (22.9%)	219 (23.0%)	< 0.001
Infrequent	241 (25.3%)	216 (22.6%)	
Sometimes	331 (34.7%)	249 (26.1%)	
Frequent	121 (12.7%)	189 (19.8%)	
Always	43 (4.5%)	81 (8.5%)	
I feel difficulty concentrating			
Never	213 (22.3%)	191 (20.0%)	< 0.001
Infrequent	347 (36.4%)	287 (30.1%)	
Sometimes	284 (29.8%)	238 (24.9%)	
Frequent	84 (8.8%)	183 (19.2%)	
Always	26 (2.7%)	55 (5.8%)	
I feel I am unable to be sensitive to the needs of coworkers and patients			
Never	308 (32.3%)	288 (30.2%)	< 0.001
Infrequent	333 (34.9%)	262 (27.5%)	
Sometimes	239 (25.1%)	256 (26.8%)	
Frequent	57 (6.0%)	128 (13.4%)	
Always	17 (1.8%)	20 (2.1%)	
Total burnout score	**18.05 ± 5.41**	**19.33 ± 6.51**	< 0.001

### Career practice before and during the COVID-19 pandemic

Although respondents reported a significant decrease in "*Participation in research work/year*" during the pandemic, there was a significant increase in "*Hours spent reading scientific articles/week*" and "*Hours working at home/week*". There was a significant reduction in both the median "*Clinical cases in outpatient clinic/week*" and "*Operative cases/week*" during the pandemic in elective practice. Conversely, there was a significant reduction in both the median "*Hours working on-call/week*" and "*Number of emergency cases/week*" in emergency practice. Overall, there was a significant reduction in both the median "*Cases working as primary surgeon/week*" and "*Cases working as assistant surgeon/week*". These details are shown in Table 3.

**Table 3:** Q 14. Career practice before and during the pandemic^a^

	**Before COVID 19 Pandemic**	**During COVID 19 Pandemic**	**P-value**
Participation in research work/year	10 (10–10)	10 (0–10)	< 0.001
Hours spent reading scientific articles/week	10 (10–20)	10 (10–20)	< 0.001
Clinical cases in outpatient clinic/week	30 (20–50)	10 (10–20)	< 0.001
Operative cases/ week	20 (10–20)	10 (10–10)	< 0.001
Cases working as primary surgeon/week	10 (10–20)	10 (0–10)	< 0.001
Case working as assistant surgeon/week	10 (10–20)	10 (0–10)	< 0.001
Hours working on-call/week	30 (10–40)	20 (10–40)	< 0.001
Hours working at home/week	10 (0–10)	10 (0–20)	< 0.001
Number of emergency case/week	10 (10–20)	10 (10–10)	< 0.001

^a^Data reported as median (percentiles; 25–75)

### Role of respondents during the pandemic and the availability of resources during the pandemic

Five questions addressed the respondent’s role during the pandemic; 81.3% reported that their hospitals were included in the management of COVID-19 cases, 66.5% felt that their role was minimized, 66.4% were in contact with COVID-19 positive cases, 41% were asked to share in non-surgical medical practice, and 37.6% were included in a COVID-19 patient management team.

Another five questions discussed the availability of resources during the pandemic, summarized in Tables 4 and 5. Respondents reported that "*surgical training resources*" were insufficient by 48.2%, neutral by 30.2%, and sufficient by 21.6%. However, they reported "*surgical research resources*" to be neutral in 38.8%, insufficient in 37%, and sufficient 24.2%. Conversely, respondents reported that "*knowledge resources regarding the pandemic*" was sufficient in 44.3%, neutral in 34.1%, and insufficient in 21.6% and that "*required skills regarding the pandemic*" was neutral in 42.5%, sufficient in 28.3%, and insufficient in 29.2%. Finally, for "*required protection during the pandemic*" respondents noted that it was insufficient in 40.8%, sufficient in 33.6%, and neutral in 25.6%.

**Table 4:** Q 15. Role During the pandemic

	**YES**	**NO**	**NA**
My hospital was included to treat COVID-19 Cases	776 (81.3%)	160 (16.8%)	18 (1.9%)
I was included in COVID-19 patient management team	359 (37.6%)	570 (59.7%)	25 (2.6%)
I was in contact with COVID-19 positive cases	633 (66.4%)	283 (29.7%)	38 (4%)
I was asked to share in medical practice away from my surgical field	391 (41%)	518 (54.3%)	45 (4.7%)
I felt my clinical role was minimized during the pandemic	634 (66.5%)	283 (29.7%)	37 (3.9%)

**Table 5:** Q 16. How do you evaluate the availability of resources during the pandemic?

	**Sufficient**	**Neutral**	**Insufficient**
Surgical training resources	206 (21.6%)	288 (30.2%)	460 (48.2%)
Surgical research resources	231 (24.2%)	370 (38.8%)	253 (37%)
Knowledge resources regarding pandemic	423 (44.3%)	325 (34.1%)	206 (21.6%)
Required skills regarding the pandemic	270 (28.3%)	405 (42.5%)	279 (29.2%)
Required protection during the pandemic	321 (33.6%)	244 (25.6%)	389 (40.8%)

## Discussion

The impact of COVID-19 on burnout on medical providers has been examined in several studies. The current study has several strengths in that it included only surgeons compared to other studies that included all healthcare workers or physicians of different specialties [8] and compared burnout among surgeons before and during the pandemic. Moreover, our study included 954 surgeons of various specialties from 65 countries with a survey completion rate of nearly 68%, which could be attributed to the power of social media combined with the incentive of co-authorship as part of the collaborative group. Finally, our results were consistent with the literature [12, 13].

As expected, there was a significant increase in burnout during the pandemic. In addition, there were significant differences for all questions except the first, "*I feel tired*". Age and years of practice showed significant negative correlations with burnout before and during the pandemic. Surgeons in surgical training programs had to overcome gaps in their training that emerged during the pandemic. Unfortunately, most healthcare systems were underprepared for such a global crisis. Despite the suspension of elective procedures, emergency surgeries were ongoing with the likelihood that personnel were operating on COVID-19 cases with scarce personal protective equipment (PPE). Another facet of burnout was the potential transmission of infection to family members, thus some surgeons may have decided to isolate from their relatives [14].

Burnout may adversely affect healthcare workers' wellbeing, which in turn adversely affects patient safety [15]. Overall, job dissatisfaction and absenteeism are well-known consequences of burnout. However, recent evidence has shown that consequences may extend to include psychical burdens such as cardiovascular diseases or musculoskeletal pain and psychological burdens such as depressive symptoms [16]. A recent meta-analysis offers evidence of significant levels of anxiety, depression, and insomnia among a total of 33,062 healthcare workers during the pandemic with the long-term impact of post-traumatic stress disorders necessitate more clarification [17, 18].

Even researchers were affected as research emphasis shifted towards COVID-19 related topics. Thus, while non-COVID-19 research efforts decreased, COVID-19 research increased yielding a not neutral effect on "*Participation in research work/week*" [19, 20]. However, there was a window of opportunity as the pandemic has improved numerous facets of biomedical trials to increase their impacts on the clinical community. Furthermore, many research opportunities with collaborative nature focusing on COVID-19 were proposed in surgery [21]. These factors and initiatives could explain the variations in "*surgical research resources*" between being neutral in 38.8%, insufficient in 37%, and sufficient 24.2%.

It seems that surgical practice was affected in almost all daily aspects of clinical practice. Our study showed a significant reduction in the median "*Clinical cases in outpatient clinic/week*", "*Operative cases/week*", and "*Hours working on-call/week*". These findings aligned with recommendations from international surgical societies for elective and emergency activities to limit viral spread and reserve all resources for COVID-19 patients. While elective activities were cancelled or postponed, emergency activities were ongoing albeit with a significant reduction in the median "*Number of emergency cases/week*" [22].

In the USA, the National Syndromic Surveillance Program reported a 42% reduction in emergency visits during the early pandemic period with a fourfold increase of infectious disease–related visits adherent with the recommendations to minimize the risk of viral transmission [23]. Unfortunately, the patients’ fear of infection combined with in-hospital logistics changed in response to the pandemic and resulted in an uneven significant delay in time-to-diagnosis and time-to-intervention with an estimated increase in more severe septic diseases [24].

The global workforce was profoundly affected by the pandemic. The International Labour Organization set a recommendation to combat the COVID-19 outbreak centered on an individual’s safety. There was a global attitude shift toward working from home [25]. Our study reported a significant increase in "*Hours working at home/week*" as surgeons could continue to conduct perioperative assessments and postoperative follow-up visits from home through telemedicine, with the added advantage of eliminating unnecessary hospital visits. Furthermore, telemedicine has emerged as a means of "forward triage" in lieu of emergency department visits [26, 27]. Virtual medical education also exploded, with hundreds of academic staff members participating daily [28].

The pandemic's profound negative impact on healthcare systems was confirmed in our study as most respondents (81.3%) reported their hospitals' inclusion in the management of COVID-19 patients, and 37.6% directly involved in their care. Furthermore, the impact on communities was evident in our study, with 66.4% of respondents reporting contact with COVID-19-positive cases. Although it took more than two months for the first 100,000 cases to be reported, in the 2 weeks prior to the start of our survey, more than 100,000 new cases were reported almost daily [29].

Approximately half (48.2%) of the respondents reported insufficient "*surgical training resources*", with a significant reduction in both the median "*Cases working as primary surgeon/week*" and "*Cases working as assistant surgeon/week*". Several unexpected factors emerged, resulting in reduced training opportunities. In an immediate response, surgical training was suspended in most countries and some trainees were deployed to serve at COVID-19 dedicated hospitals or areas within hospitals. Based on international recommendations, most elective non-cancer procedures were cancelled or postponed, cancer and transplant procedures were allocated to specific centers, telemedicine replaced the usual patient office visits, and academic activities including conferences, symposia, and workshops were cancelled or organized virtually [7, 30, 31].

The significant increase in "*Hours spent reading scientific articles/week*" may have been multifactorial. Specifically, time spent at home and increased availability of online education were both major factors. The critical shortage of PPE was a demanding situation attributed to the problems with the global supply chain and this could be reflected in our study as 40.8% of the respondents found that the required PPEs were insufficient [32].

### Limitations

The findings of this study should be taken in the context of certain limitations. First, the design is cross-sectional with a convenience sample, self-report, and possibly nonresponse bias, limiting its generalization. Second, the study was administered during the COVID-19 pandemic and responses may vary in different geographical regions related to infection or even in future waves. Finally, other unmeasured factors may account for variations in burnout.

Future studies should further explore the prevalence, consequences, and appropriate intervention to mediate the effects of burnout using probability samples with longitudinal designs. This should be combined with increased awareness of burnout to help complete such surveys. Resources should be directed at better understanding the risk factors, identifying early signs, and supporting those at high-risk, combined with strategies to improve the workforce environment. Measures should be undertaken to offer equal training opportunities even under these difficult situations, and new avenues of surgical training need to be explored.

## Conclusion

There was a significant burnout among trainees magnifying the protective role of longer years of practice and older age. Almost all aspects of clinical and research activities were affected with significant reduction in the number of research work undertaken, outpatient clinic visits, surgical procedures, on-call hours, and emergency surgery cases, which could hinder training opportunities. The majority of respondents felt that their role was minimized with insufficient surgical training resources and inclusion in COVID-19 non-surgical management. Finally, more than one third of respondents felt they had insufficient protection during the pandemic.

### Supplementary Information

**Additional file 1.**

**Additional file 2.**

## Data Availability

The corresponding author will provide any information about the data presented in the article when requested.

## References

[1] Melamed S, Kushnir T, Shirom A (1992). Burnout and risk factors for cardiovascular diseases. Behav Med (Washington, DC).

[2] Gerber M, Colledge F, Mücke M, Schilling R, Brand S, Ludyga S (2018). Psychometric properties of the Shirom-Melamed Burnout Measure (SMBM) among adolescents: results from three cross-sectional studies. BMC Psychiatry.

[3] Oskrochi Y, Maruthappu M, Henriksson M, Davies AH, Shalhoub J (2016). Beyond the body: A systematic review of the nonphysical effects of a surgical career. Surgery.

[4] Balch CM, Freischlag JA, Shanafelt TD (2009). Stress and Burnout Among Surgeons: Understanding and Managing the Syndrome and Avoiding the Adverse Consequences. Arch Surg.

[5] WHO. Burn-out an "occupational phenomenon": International Classification of Diseases: WHO; 2019. News]. Available from: https://www.who.int/news/item/28-05-2019-burn-out-an-occupational-phenomenon-international-classification-of-diseases. [Cited 12th Mar 2021].

[6] WHO. WHO Coronavirus (COVID-19) Dashboard: WHO; 2021. Available from: https://covid19.who.int/ [Cited 12th March 2021]

[7] Daodu O, Panda N, Lopushinsky S, Varghese TK Jr, Brindle M. COVID-19 - Considerations and Implications for Surgical Learners. Ann Surg. 2020;272(1):e22-e23. 10.1097/SLA.0000000000003927. 10.1097/SLA.0000000000003927 32345789

[8] Sharifi M, Asadi-Pooya AA, Mousavi-Roknabadi RS (2020). Burnout among Healthcare Providers of COVID-19; a Systematic Review of Epidemiology and Recommendations. Arch Acad Emerg Med.

[9] Eysenbach G (2004). Improving the quality of Web surveys: the Checklist for Reporting Results of Internet E-Surveys (CHERRIES). J Med Internet Res.

[10] Melamed S, Ugarten U, Shirom A, Kahana L, Lerman Y, Froom P (1999). Chronic burnout, somatic arousal and elevated salivary cortisol levels. J Psychosom Res.

[11] World Bank Country and Lending Groups: World Bank 2021. Available from: https://datahelpdesk.worldbank.org/knowledgebase/articles/906519-world-bank-country-and-lending-groups. [Cited 10th April 2021].

[12] Aziz H, James T, Remulla D, Sher L, Genyk Y, Sullivan ME (2020). Effect of COVID-19 on Surgical Training Across the United States: A National Survey of General Surgery Residents. J Surg Educ.

[13] Pertile D, Gallo G, Barra F, Pasculli A, Batistotti P, Sparavigna M (2020). The impact of COVID-19 pandemic on surgical residency programmes in Italy: a nationwide analysis on behalf of the Italian Polyspecialistic Young Surgeons Society (SPIGC). Updat Surg.

[14] Blanco-Colino R, Soares AS, Kuiper SZ, Zaffaroni G, Pata F, Pellino G (2020). Surgical Training During and After COVID-19: A Joint Trainee and Trainers Manifesto. Ann Surg.

[15] Hall LH, Johnson J, Watt I, Tsipa A, O’Connor DB (2016). Healthcare Staff Wellbeing, Burnout, and Patient Safety: A Systematic Review. PLoS ONE.

[16] Salvagioni DAJ, Melanda FN, Mesas AE, González AD, Gabani FL, Andrade SM (2017). Physical, psychological and occupational consequences of job burnout: A systematic review of prospective studies. PLoS ONE.

[17] Pappa S, Ntella V, Giannakas T, Giannakoulis VG, Papoutsi E, Katsaounou P (2020). Prevalence of depression, anxiety, and insomnia among healthcare workers during the COVID-19 pandemic: A systematic review and meta-analysis. Brain Behav Immun.

[18] Tan YQ, Wang Z, Yap QV, Chan YH, Ho RC, Hamid ARAH, et al; SoMe4Surgery working group Collaborators. Psychological Health of Surgeons in a Time of COVID-19: A Global Survey. Ann Surg. 2023;277(1):50–6. 10.1097/SLA.0000000000004775. 10.1097/SLA.0000000000004775 PMC9762613 33491983

[19] van Dorn A (2020). COVID-19 and readjusting clinical trials. The Lancet.

[20] Bierer BE, White SA, Barnes JM, Gelinas L (2020). Ethical Challenges in Clinical Research During the COVID-19 Pandemic. J Bioeth Inq.

[21] Collaborative C (2020). Mortality and pulmonary complications in patients undergoing surgery with perioperative SARS-CoV-2 infection: an international cohort study. Lancet.

[22] Moletta L, Pierobon ES, Capovilla G, Costantini M, Salvador R, Merigliano S (2020). International guidelines and recommendations for surgery during Covid-19 pandemic: a systematic review. Int J Surg (London, England).

[23] Reichert M, Sartelli M, Weigand MA, Doppstadt C, Hecker M, Reinisch-Liese A (2020). Impact of the SARS-CoV-2 pandemic on emergency surgery services-a multi-national survey among WSES members. World J Emerg Surg.

[24] Hartnett KP, Kite-Powell A, DeVies J, Coletta MA, Boehmer TK, Adjemian J, et al. Impact of the COVID-19 Pandemic on Emergency Department Visits — United States, January 1, 2019–May 30, 2020 USA: Centers for Disease Control and Prevention; 2021. Available from: https://www.cdc.gov/mmwr/volumes/69/wr/mm6923e1.htm?s_cid=mm6923e1_w#suggestedcitation. [Cited 18th March 2021].

[25] ILO. An employers’ guide on working from home in response to the outbreak of COVID-19: International Labour Organization 2020. Available from: https://www.ilo.org/actemp/publications/WCMS_745024/lang--en/index.htm. [Cited 18th Mar 2021].

[26] Kichloo A, Albosta M, Dettloff K, Wani F, El-Amir Z, Singh J (2020). Telemedicine, the current COVID-19 pandemic and the future: a narrative review and perspectives moving forward in the USA. Fam Med Community Health.

[27] Asiri A, AlBishi S, AlMadani W, ElMetwally A, Househ M (2018). The Use of Telemedicine in Surgical Care: a Systematic Review. Acta Inform Med.

[28] Favale T, Soro F, Trevisan M, Drago I, Mellia M (2020). Campus traffic and e-Learning during COVID-19 pandemic. Comput Netw.

[29] WHO. WHO Director-General's keynote speech at the T20 Task Force: Policy Recommendations for a Post-COVID-19 World- 15 June 2020 Geneva, Switzerland: World Health Organizaton 2020 [cited 2021 Februay 28th]. Available from: https://www.who.int/director-general/speeches/detail/who-director-general-s-keynote-speech-at-the-t20-task-force-policy-recommendations-for-a-post-covid-19-world--15-june-2020.

[30] COVIDSurg Collaborative. Elective surgery cancellations due to the COVID-19 pandemic: global predictive modelling to inform surgical recovery plans. Br J Surg. 2020;107(11):1440–9. 10.1002/bjs.11746. 10.1002/bjs.11746 PMC7272903 32395848

[31] ACPGBI Legacy Working Group. Legacy of COVID-19 - the opportunity to enhance surgical services for patients with colorectal disease. Colorectal Dis. 2020;22(10):1219–28. 10.1111/codi.15341. 10.1111/codi.15341 32857886

[32] Ranney ML, Griffeth V, Jha AK. Critical Supply Shortages - The Need for Ventilators and Personal Protective Equipment during the Covid-19 Pandemic. N Engl J Med. 2020;382(18):e41. 10.1056/NEJMp2006141. 10.1056/NEJMp2006141 32212516

