# Correction: Burnout among surgeons before and during the SARS-CoV-2 pandemic: an international survey

**DOI:** 10.1186/s40359-024-01577-0

**Published:** 2024-03-01

**Authors:** Mostafa Shalaby, Ahmed M ElSheikh, Hosam Hamed, SURG-S. A. T.-19 Collaborative Group

**Affiliations:** 1 Department of General Surgery, Mansoura University, 60 ElGomhouria Street, Mansoura, 35516 Dakahliya Egypt; 2 Department of Quality and Patient Safety, Security Forces Program Hospital Makkah, Makkah, Kingdom of Saudi Arabia

**Correction: BMC Psychology 12, 48 (2024)**

**https://doi.org/10.1186/s40359-023-01517-4**

After publication of this article [1], the authors reported that the author’s name Ahmed M. Elsheikh was incorrectly written as Ahmad Elsheik.

The original article has been corrected.

## References

[1] Shalaby M (2024). Burnout among surgeons before and during the SARS-CoV-2 pandemic: an international survey. BMC Psychology.

