# Supplementary material for: Burnout among surgeons before and during the SARS-CoV-2 pandemic: an international survey
Source: BMC Psychol. 2024 Jan 25;12:48. doi: 10.1186/s40359-023-01517-4 (PMC10810224; doi:10.1186/s40359-023-01517-4)
Supplement: Supplementary file 1 — Additional file 1. [file 40359_2023_1517_MOESM1_ESM.docx]

**Supplementary Material Members of the SURG-SAT 19 Collaborative**

**Survey development:** Hosam Hamed and Mostafa Shalaby

**Survey revision:** Ahmed Elsheik

**Pretesting group:** Ahmad Sakr, Amgad Fouad, Amr Kassem, Hossam Elfeki, Khaled Madbouly, Khalid H Alzahrani, Khalid Marzouk, Mahmoud Ali, Mohamed Alaa Abdelmoez Helal, Mohamed Elsorogy, Mohamed Farid, Nicola Di Lorenzo, Pierpaolo Sileri, Steven Wexner, Wael Khafagy

**Project steering:** Ademola Adeyeye, Alaa El-Hussuna, Alice Frontali, Avanish Saklani, Benedettao Lelpo, Daniela Molena, Diwakar Pandey, Elena Karbovnichaya, Francesco Pata, Gabrielle H. Van Ramshorst, Gaetano Gallo, Gaya Spolverato, Gianluca Pellino, Giulia Bagaglini, Ines Rubio-Perez, Ionut Negoi, Isabella Frigerio, Jovan Juloski, Khalid H Alzahrani, Marijana Ninkovic, Marzia Franceschilli, Mina Azer, Sergey Efetov, Simona Ippoliti, Steven Wexner, Zoe Garoufalia

**Writing group:** Mostafa Shalaby, Hosam Hamed, Ahmed Elsheik, Alaa El-Hussuna, Gabrielle H. Van Ramshorst, Gaetano Gallo, Gianluca Pellino, Ionut Negoi, Steven Wexner

**Statistical analysis:** Mostafa Shalaby, Hosam Hamed, Ahmed Elsheik

**Data Handling:** Ahmed Elsheik

**Collaborators (SURG-SAT 19): Country based**

**Afghanistan:** Mohammad Rafi Fazli

**Albania:** Agron Dogjani

**Algeria:** Harieche Abdennour, Abderahim Cherfa, Tilioua Omar

**Argentina:** Javier Minoldo, José Maria Alvarez Gallesio, Matias Quesada

**Austria:** Annica Bacher, Stephan Kropshofer, Florian Ponholzer, Philip Tesik, Philipp Gehwolf, Sevim Isci, Stefan Uranitsch, Valeria Berchtold

**Azerbaijan:** Elgun Samadov

**Bahrain:** Abdulmenem Abualsel

**Bangladesh:** Ashrarur Rahman Mitul, S. M. Nazmul Islam

**Belgium:** Aude Vanlander, Charles Van Praet, Elke Van Daele, Hanne Vanommeslaeghe, Jasper Stijns, Kessewa Abosi-Appeadu, Martijn Depuydt, Mathias Allaeys, Van Nieuwenhove Yves

**Brazil:** Ramiro Colleoni

**Bulgaria:** Mihail Slavchev

**Canada:** Aly Elbahrawy, Jessica G.Y. Luc, Karen Milford

**Croatia:** Ivan Romic

**Denmark:** Alessio Monti, Ashraf Haydal, Mads Falk Klein, Miranda E. K. Ocklind, Sabah Anwar Hadi

**Egypt:** Abdallah Alqasaby, Abdelazim Elganash, Adel Goda Hussein Daibes, Adham Elsaied, Ahmad Elhattab, Ahmad Lotfy, Ahmed Alnashar, Ahmed Abd Elbaset Elsayed Abu Elnour, Ahmed Abdelhalim, Ahmed Abdelhamid, Ahmed Abdellatif, Ahmed Abdelmohsen, Ahmed Abdelrafee, Ahmed Adel Elhawary, Ahmed Azmy Zidan, Ahmed Eleshra, Ahmed Elkafoury, Ahmed Ezz, Ahmed Ezzat Elghrieb Abdelmomen, Ahmed Farag Elkased, Ahmed Fawzy, Ahmed G Elkhouly, Ahmed Gamal Abouelfetouh Ibrahim Hemidan, Ahmed Hosam Eldin Hasan Abbas, Ahmed Mahmoud Ahmed Ismail, Ahmed Mohamed Attia, Ahmed Mohammed Farid, Ahmed Mostafa Elnakash, Ahmed Negida, Ahmed Soliman, Ahmed Taki-Eldin, Ali Almahdy Ali Albadry, Aly Sanad, Amira Alsayed Abdelhai Elbatal, Amr Elgazar, Amr Saleh, Andrew Fahiem, Anwar Yahya A Mohamed, Ashraf Nageeb, Ashraf S. Elmetwally, Ayman Alkhalegy, Ayman El-Wakeel, Ayman Shemes, Bashir A. Fadel, Basma Waseem Lutfi, Doaa Ali, Khaled Samir Abolnasr, Ehab Gamal, Emad Abdallah, Emad Ali Ahmed, Eman Abdalla Mohamed Salem, Esmael Ali Hamed, Essam Elshikh, Farazdaq Enad, Fetoh Alaaeldin Fetoh Sarhan, Galal Abouelnagah, Gamal Hassan El Tagg, Gehad Atef, George Samir Habib Shaker, Hatem Beshir, Hazem M Zakaria, Hesham Barbary, Hesham Elgendy, Hesham Sharaf, Hisham Elnaghi, Hosam Elghadban, Ibrahim Elzayat, Ibrahim Fakhr, Ibrahim Sallam, Ibrahim Tharwat Mohamed Abdelmoneim, Islam Elnemr, Karem Shahin Mohamed Zewar, Khaled Elalfy, Khaled Sabet, Khaled Yousery Ibrahim Mansour, Khalid Abdalla Abdelgadir Osman, Maher Elesawi Kamel Elgaly, Maher Shams, Mahmoud Abozeid, Mahmoud M Mohammed, Mahmoud Mohamed Elkatt, Mahmoud Yahia Samaha, Marolla Maher Eskander Mikhael, Medhat M H A Khalil, Moaaz Alhendawey, Mohamad Elrefai, Mohamed A. Gabr, Mohamed Abdelaziz Mohamed Abdalla Mohamed Ali Fayed, Mohamed Abdelmaksoud, Mohamed Abouelmagd Salem, Mohamed Adel Mohamed Mohamed, Mohamed Adel Nabeeh, Mohamed Ahmed Abdelhalim Ahmed Elsayed, Mohamed Ahmed Abdelmonem, Mohamed Anwar Abdel Razik Ali, Mohamed Eldemery, Mohamed Elmesery, Mohamed Fikry, Mohamed Gharbia, Mohamed I Omar, Mohamed Ibrahim Elmoghazy, Mohamed Jomma Ghazala, Mohamed Korayem Fattouh Hamed, Mohamed Metwally, Mohamed Mohamed Hamdy Arnouse, Mohamed Mohsen Amen, Mohamed Mokhtar Amary, Mohamed Mosaad Kandel, Mohamed Mostafa Abuzeid, Mohamed Rabea, Mohamed Ramadan Sobh, Mohamed Taman, Mohammad Fathy, Mohammad Montaser Hassan Moustafa, Mohammad Zuhdy, Mohammed Adel, Mohammed Alaa, Mohammed Alawady, Mohammed El Edassy, Mohammed Mustafa Hassan Mohammed, Mohammed Nabil Eldesouki, Mohammed Said Mahmoud Salim, Mohammed Sanad, Mohsen George Khalaf, Mohsen Michael Henes, Momen Abdelglil, Mona Mhmoud Mohmmed, Morsi Mohamed Morsi Abdelkhalik, Mosab Shetiwy, Mostafa Elshazli, Mostafa Hegazy, Mostafa Mahmoud Ahmed, Mostafa Mohammed Abdelhalim, Mostafa Shahein, Mostafa Sofan, Muhammed Alaa Moukhtar Hammad, Mustafa Ahmad, Nader Milad, Nehal Farouk, Omnia Eldesouky, Omnia Y Mohamed, Osama Abdel Salam Mahadel, Osama Gaarour, Radwan Abdelsabour Torky, Raheem El-Gohary Abd Elhafez, Ramy Magdy Adly, Ramy Mikhael Nageeb, Salah Hamdi, Sameh Gamal, Sameh Hany Emile, Samer Regal, Sayed Abdelrasheed, Shady Ahmed Elzeftawy, Sohib Mohammed Mohammed Khashshan, Tamer Ashraf, Tamer Khafagy, Tamer Nabil, Tarek Abdelazim, Tarek Taher Rizk, Wesam Amr, Yousef Mohamed Yousef, Youssef Abdel Aziz Youssef

**France:** Antonio Castaldi, Antonio Fiore, Ariola Hasani, Aurora Mariani, Claire Dagorno, D'Alessandro Antonio, Giuliano Izzo, Giulio Addari, Giuseppe Mangiameli, Lo Dico Rea, Luca Pio, Marco Paci, Police Andrea, [G Serena De Fatico](https://pubmed.ncbi.nlm.nih.gov/?size=200&term=De+Fatico+GS&cauthor_id=26708852), Tartaglia Elvira

**Germany:** Alejandro Daniel Lira Schuldes, Eslam Rihan, Gabriela Moeslein, Hans Lederhuber, Ibram Botros, Ismail Jaman, Johannes Doerner, John Rezk Hanna Elseberbihy, Kareem El Sherbiny, Mostafa Ghonim, Amir Mikrish, Mina Aziz, Mohamed Hatm, Rami Archid, Samuel Elkess Morcos Gendy, Sufian Ahmad

**Greece:** Alexandros Charalabopoulos, Anastasia Prodromidou, Argyrios Ioannidis, Eustratia Mpaili, Garyfallia Boukorou, Georgios Papadopoulos, Theodore Liakakos, Vasileiadou Styliani

**India:** Abhishek Agrawal, Amita Jain, Arshad Rashid, Asif Mehraj, Swagata Brahmachari, Harish Neelamraju Lakshmi, Kushagra Vishwakarma, Lalit Parida, Meenakshi Sharma, Mohammad Zaieem, Murtaza Makasarwala, Rigved Nittala, Sanjeev Kumar, Sharma Vikrantmr, Sheikh Junaid, Somyaa Khuller, Vinal More

**Iraq:** Abeer Abdul Hameed Ahmed, Adil Alomieri, Arkan Shubber Alhamdany, Muslim Ka Del, Ghadah Najm, Nawras Falah Lateef

**Ireland:** Deborah Mcnamara, Mohammed Elkassaby Abdelmageed, Mudassar Majeed

**Italy:** Albert Troci, Alberto Porcu, Alessandra Marano, Alessandro Di Bartolomeo, Alessandro Giani, Alessandro Giardino, Alfonso Canfora, Andrea Balla, Andrea Barberis, Andrea Belli, Andrea Borasi, Andrea Manetti, Andrea Mingoli, Andrea Morini, Angela Maurizi, Angelo Alessandro Marra, Angelo Gabriele Epifani, Angelo Iossa, Angelo Parello, Anna Guida, Anna Maffioli, Anthony Kevin Scafa, Antonino Spinelli, Antonio Matarangolo, Arcangelo Picciariello, Brunella Pirozzi, Bruno Cirillo, Carlo Gazia, Carlo Ratto, Caterina Foppa, Chiara Marafante, Chierici Andrea, Cinzia Tanda, Claudio Guerci, Cristine Don, Daniele Zigiotto, Denise Coniglio, Diego Sasia, Diego Visconti, Donato F Altomare, Eleonora Guaitoli, Emanuele Botteri, Enrico Pinotti, Fabio Martinelli, Fabio Uggeri, Fabrizio Bàmbina, Federica Falaschi, Federico Costanzo, Filippo La Torre, Flavio Milana, Francesca Abbatini, Francesca De Lucia, Francesca Paola Tropeano, Francesco Colombo, Francesco Ferrara, Francesco Litta, Francesco Maria Carrano, Francesco Orlando, Francesco Roscio, Francesco Selvaggi, Gabriella Giarratano, Gianluca Pagano, Giorgio Lisi, Giulio Argenio, Giuseppa Zancana, Giuseppe Cavallaro, Giuseppe Frazzetta, Grasso Mariateresa, Guido Sciaudone, Ivan Vella, Leandro Siragusa, Letizia Santurro, Lorenzo Ferri, Lorenzo Petagna, Luca Ferrario, Ludovica Pitoni, Marcello Filograna Pignatelli, Marco Angrisani, Marco Giugliano, Marco Inama, Marco V. Marino, Marco Veltri, Maria Carmela Giuffrida, Maria Paola Menna, Marina Valente, Matteo Rottoli, Matteo Sacchi, Matteo Uccelli, Maurizio Rho, Mauro Garino, Mauro Montuori, Michela Campanelli, Monica Zese, Nadia De Falco, Nicola Cillara, Nicolò Maria Mariani, Nicolò Tamini, Ottavio Adorisio, Paola Campennì, Paolina Venturelli, Paolo Bernante, Paolo Sapienza, Pasquale Cianci, Patrizia Marsanic, Pierfrancesco Lapolla, Piero Tecchio, Pietro Familiari, Pietro Fransvea, Placido Bruzzaniti, Redan Hassan, Riccardo Pirovano, Roberto Rimonda, Salomone Di Saverio, Sara Di Carlo, Teresa Perra, Tommaso Campagnaro, Valentina Testa, Valeria Andriola, Virgilio Michael Ambrosi Grappelli, Vita Capizzi, Vito Chiarella, Vittoria Bellato

**Japan:** Katsuhiko Yanaga, Mohamed Farouk

**Jordan:** Ahmad Uraiqat, Mahmoud Almasri

**Kenya:** Ambrose Nabwana, Mark M. W. Siboe, Njoroge P. W, Githu Njoroge, Jh. Ilkul, Ralph Ombati Obure, Yusuf Palkhi

**Kuwait:** Ali Alkhayat, Ali Sayed Ali, Amgad Nashaat Abdel Malek, Emad Fahim Abdelsayed, Tarek Zahra

**Lebanon:** Larissa Ayoub, Fadi Sleilati, Rany Aoun

**Libya:** Nassib Algatanesh, Nura Ahmed Fieturi

**Malaysia:** Jen Siang Ng

**Mexico:** Andrés Vega Díaz, Erik Efrain Sosa Duran, José Eaazim Flores Guerrero, Manuel Meza Jasso, Manuel S Salas Flores, Marcos José Serrato Felix, Victor Manuel Pinto Angulo

**Morocco:** Abdelhadi Mejdane, Abdelmounaim Aitali, Benzakour Amal, Aziz Zentar, Ahmed Bensaad, El Alami Yacir, Fassi Fihri Mohamed Jawad, Mohamed Ghassane Rachid, Mohamed Maliki-Alaoui, Mouaqit Ouadii, Ouazni Mohammed

**Myanmar:** Nyan Thein

**Nepal:** Dinesh Prasad Koirala

**Netherlands:** Denise Hilling, Sjaak Pouwels

**Nigeria:** Abiodun Idowu Okunlola, Adeyinka Adejumo, Akinola Akinmade, Asimiyu Adekunle Shittu, Ayodele Samuel Oluyomi, Azeez Lateef Abiodun, Bashir Lawal, Clement Odion, Ademola Popoola, Edward Jolayemi, El-Zaki Shomoye, Funmilola Olanike Wuraola, Grace Eke, Henry Abiyere, Ige Oluwasuyi, Ihediwa George, Iloba Gabriel Njokanma, Isiaka Aremu, Julius Kolajo Dare, Lukman Abdur-Rahman, Misbahu Haruna Ahmad, Mobolaji Adewale Oludara, Mohammad Aminu Mohammad, Ojajuni Adeoluwa, Oladele Situ, Peter Agbonrofo, Raji Taofiq Kewulere, Yakubu Aliyu, Yusuf Adebowale

**Oman:** Ahmed Galala, Satish Rao

**Pakistan:** Aasma Waleed, Aatif Inam, Abdul Razaque Shaikh, Ahmad Uzair Qureshi, Aneeqah Din Muhammad, Arooj Ahmed, Asad Ali Kerawala, Mohammad Aslam, Asma Mehr, Ayesha Javed, Farooq Ahmad, Haroon Javaid Majid, Hassan Ahmed, Irfan Daudi, Khalid Akhtar, Khurram Niaz, Mariyah Anwer, Mohammed Amir, Muhammad Amir Hanif, Muhammad Asif, Muhammad Asif Raza, Muhammad Imran Khokhar, Muhammad Khurram Jameel, Muhammad Nasir, Muhammad Salman Shafique, Mujammad Ateeb, Munawar Nadeem, Rahmat Ullah Shah, Shahzad Hussain Waqar, Shahzad Alam Shah, Talat Waseem, Tariq Ghafoor, Tauseef Fatima, Umar Bashir

**Peru:** Erick Ivan Huaman Gonzales, Luis Angel Garcia Ruiz

**Portugal:** Carla Freitas, Xavier De Sousa

**Qatar:** Ahmed Al-Bahrani, Carlos Antonio Sanchez Portela, Elsayed Aly Elgazar, Eloy Morasen Robles, Irfan Jan Khan, Lutfi Jarboa, Mahwish Khawar, Miguel Jose Pinto Echevarria, Moataz M Bashah, Salahaldeen Dawdi, Shameel Musthafa, Syed Muhammad Ali

**Romania:** Cezar Ciubotaru, Eduard-Alexandru Bonci, Mihai-Stefan Muresan, Stoica Bogdan, Tanase Ioan

**Russian Federation:** Albina Zubayraeva, Aleksandr Derinov, Alexander Zakharenko, Anastasia Novikova, Andrey Bashlachev, Ayrat Kaldarov, Berelavichus Stanislav, David Gorin, Dmitriy Puzenko, Ekaterina Kazachenko, Erkin Ashimov, Iuliia Medkova, [Ivan Ignatov](mailto:ignatov.mac93@mail.ru), Kochetkov Viktor Sergeevich, Lyudmila Sidorova, Michail Kiselev, Michail Danilov, Ogoreltsev Aleksandr, Sergey Rodimov, Tatiana Garmanovs, Yury Kitsenko, Nekoval Valery

**Rwanda:** Ntezamizero Japhet

**Saudi Arabia:** Abdulrahman Sibiany, Abdelhalim Saadeldin, Abdelrahman Abuosba, Abdulbari Mohammed Alawadhi, Abdulhamid Alharbi, Abdullah Althumali, Abdullah Alghuliga, Abdullah Alotaibi, Abdullah Fayez Abduraboh, Abdullah Kateb, Abdullah Sindy, Abdulmohsen Al Eisa, Abdulrahman Alotaibi, Abdulrhman Almulhim, Adel Ali Aljawhari, Ahmad Mahmoud Abozeid, Ahmad Saad, Ahmed Alqarni, Ahmed Alwan, Ahmed Alwusaibie, Ahmed Bafaraj, Ahmed Eldeeb, Ahmed Tarabay, Mahfoudh Mohammed, Alhanouf Alhedaithy, Alhassan Hesham Almaghrabi, Ali Ibrahim Eldawy Abed, Alqahtani Ali Abdullah, Anmar Semilan, Mohamed Farag, Essa Khudhayr, Marwah Hussain, Ghanem Abbas, Heba Alqudaihi, Abdulrahman Alotaibi, Yousra Abualnaja, Abelnasser Shaheen, Ashraf Abdelazeem Mohamed Mubarak, Bandar Idrees A Ali, Barrag Alhazmi, Bilal Ahmed Hijazi, Chadi Abdulrahman, Charles Olajide Oyedepo, Heythem Alzamel, Elsanousi Ibrahim Sabir Tairab, Munir A Alsuwaimel, Soha Hejazi, Emad Alnoqaidan, Fade Ahmed Alhussien, Fadi Sami Jallad, Faisal Khadwardi, Faisal Saleh Alghamdi, Feras Haddad, Fozan Sauri, Haitham Alafghani, Haitham Alfalah, Hamada Gad, Hamdy Haggag Ebrahim Aboelmagid, Hamed Ibrahim, Hany M Elzayady, Hatem Abdelrahman Ahmed Sharafeldin, Hatem A. Sembawa, Haytham Alabbas, Hazem Abbas, Hesham Elgamal, Homoud Alawfi, Humood Al-Sadery, Hussien Ali Abdelmotaleb, Ibrahim Al Hassn, Ishag M. Mudawi, Islam Nekhala, Kareem Elsanhoury, Khalid Babieker Said, Khalid A Albeshri, Khalid Albahooth, Khalid Fathelrahman Bakier Mohammed, Khalid Mohammad Ibrahim Asar, Luqman Osman, Mahdi Alzamanan, Mahmoud Alnabarawi, Majid Althobaiti, Mohamed Abdelmoneim Elsayed, Mohamed Al Naeb, Mohamed Salah Eldin Hassan, Mohamed Sayed Abdelhamid, Mohammad Alyami, Mohammad Amin Mirza, Mohammad Sayouh, Mohammed Amer Alkhayat, Mohammed Basendowah, Mohammed Ghunaim, Mohammed Khalid Alhussaini, Mohammed Khoj, Mohammed Sbaih, Muhammad Ahmad Saeed, Muhammad Zulfiqar Ali, Nabil Yassin Tammam Abdelaziz, Nadim Malibary, Nael Abdo, Nasser Mohammed Amer, Neamat Ahmed Ali Al Turki, Norah Durayb, Nouf Yassin, Nouf Akeel, Noureddine Larbi, Ofays Alsallum, Omar A Abu Suliman, Osama Elsherbiny, Osama Abusalem, Ibrahim Altedlawi Albalawi, Raid Abdullah Abutalib, Rayan Alarabi, Roaa Ghazi Khan, Saleh Alazzam, Saleh Alghamdi, Salem Alsawat, Sami Salim, Sarah Alshukr, Saud Alzahrani, Smain Golea, Tumadher Alowairdhi, Usama Salman, Wael Abusiam, Wael Abualkhair, Wael Saber, Wail Tashkandi, Waleed Alhazmi, Waleed Tashkandi, Wassim Abou Yassine, Yaser Ahmad Alshabi, Yaser Ibrahim, Yasser Shahin, Yassin Ibrahim, Yousef Aljathlany, Yousef Alnahas, Yousef Alrashidi, Zubair Wali

**Senegal:** Abdourahmane Ndong, Mamadou Ba, Papa Mamadou Faye

**Serbia:** Dragana Arbutina, Ljiljana Milic, Vladica Cuk

**Somalia:** Abdinafic Mohamud Hussein

**South Africa:** Jeannie Mccaul, Laurie Bertels, Linda Pohl, Marion Arnold, Nomonde Mbatani, Pj Oosthuizen, Shreya Rayamajhi, Susan Vosloo, Uzair Jooma

**Spain:** Aitor Landaluce-Olavarria, Alba Vázquez-Melero, Alberto Marcos, Alejandro Puerto Puerto, Alicia Ruiz De La Hermosa, Ana Senent-Boza, Bakarne Ugarte-Sierra, Beatriz Cros Montalbán, Beatriz Martin-Perez, Caroina Gonzalez Gomez, Enrique Colás-Ruiz, Esther Garcia Santos, Fatima Senra, Ismael Mora-Guzmán, Jana Dziakova, Jeancarlos J. Trujillo Díaz, Jesús Silva, Juan Luis Blas Laina, Luis Tallon-Aguilar, Marcello Di Martino, Mario Franco Chacón, Matteo Frasson, Mikel Prieto Calvo, Monica Millan, Patricia Tejedoe, Sonia Pérez-Bertólez, Víctor Turrado-Rodríguez

**Sudan:** Abdelrhman Azhari Mohammed Elsanosi, Duaa Abdalbakheet, Mohamed Ahmed, Omer El Faroug H Salim

**Sweden:** Mohamed Youssef

**Switzerland:** Carlotta Barbon

**Tunisia:** Amal Bouchrika, Houcine Maghrebi, Issam Loukil

**Turkey:** Alp Yildiz, Ayberk Dursun, Baris Gulcu, Bulent Calik, Burak Eral, Değercan Yeşilyurt, Fatih Yakar, Furkan Atakan Akin, Gizem Kilinc, Gülberk Uslu, Korhan Tuncer, Mehmet Ali Koc, Sezai Leventoğlu, Selman Sokmen, Semra Demirli Atici, Tayfun Kaya, Ümit Akın Dere, Yasemin Kırmızı

**Uganda:** Kavuma Daniel Ssenono, Herman Lule, Ronald Mbiine

**United Arab Emirates:** Ahmed Hamza, Shabeer Ali, Saidalavi Padinhare Peediyakkal, Gopala Pillay Varma, Haidar Aal Mussa, Hayder Makki Al-Masari, Mina Shehata, Moham Seiam, Muhammad Akram Abdul Aziz, Nessrein Nimir, Ritu Khare, Shahid Rashid, Shuiab Kazim, Zafar Gondal

**United Kingdom of Great Britain and Northern Ireland:**

Ahmed Elshawadfy Sherif, Ahmed Ghanem, Ahmed Hazem I Helmy, Ahmed Ibrahim, Ahmed Mohammed Elshaer, Ahmed Msm Marzouk, Alessandro Paolo Tamburrini, Alessandro Parente, Alexander Light, Angela Diamantopoulou, Baljit Singh, Binay Gurung, Claire Frauenfelder, Cosimo Alex Leo, Dimitri Raptis, Dixa Thakrar, Thumuluru Kavitha Madhuri, Efthymia Tsounaki, Emanuele Garreffa, Fiammetta Soggiu, George Stavrou, Hwei Jene Ng, Hani Tabasi, Hazem Nasef, Ioannis D. Kostakis, James Jeffery, Janindra Warusavitarne, Jon Lund, Kamran Qurashi, Kapil Sahnan, Kin Seng Tong, Luca Orecchia, Mandeep Kaur, Mariam Zaidi, Mario Ganau, Mohamed Ali Gad Hassan, Nathan Curtis, Nikita Bhatt, Nikolaos Machairas, Noman Zafar, Omar Toma, Panchali Sarmah, Majid Bassuni, Justin Davies, Sami Shawer, Sherif Shawer, Sophia Lewis, [Sivaraman](https://pubmed.ncbi.nlm.nih.gov/?size=200&term=Subramanian+S&cauthor_id=20056058) Subramanian, Suhaib Ahmad, Uqba Nadeem

**United Republic of Tanzania:** Aidan Njau

**United States of America:** Aley Eldin Tohamy, Andrea M. Pakula, Andrea Simioni, Bennie L. Jarvis, Georgios P. Skandalakis, Hosai Todd Hesham, Isaac A. Isaiah, Jennifer Villwock, Linda W Martin, Melissa Kress, Merry Sebelik, Sanaz Lathan, Shirin Towfigh, Stefan D. Holubar, Steve Demeester

**Yemen:** Mohammed Mohammed Hasan Alshehari, Saif Ali Ghabisha, Shehab Ahmed Ali Abdulatef, Waheeb Al-Kubati, Yasser Abdurabo Obadiel

**Zambia:** Alexander Gots, Mildred Nakazwe, Jackson Chipaila

**Zimbabwe:** Dennis Mazingi
