# Supplementary material for: Burnout among surgeons before and during the SARS-CoV-2 pandemic: an international survey
Source: BMC Psychol. 2024 Jan 25;12:48. doi: 10.1186/s40359-023-01517-4 (PMC10810224; doi:10.1186/s40359-023-01517-4)
Supplement: Supplementary file 2 — Additional file 2. [file 40359_2023_1517_MOESM2_ESM.pdf]

Effect of COVID19 Pandemic on Surgeons' Job Satisfaction and Burnout

Your participation is very valuable

Dear Surgeon, the primary purpose of this survey is to measure the effect of COVID 19 pandemic on the surgeons' satisfaction and burnout; this will help to understand the key drivers of satisfaction.

Participation in this survey is entirely voluntary, and the information collected will be anonymous, strictly confidential, and help institutes to plan better.

Any participant who will complete the Survey will be listed under "Surg-SAT-19 collaborators" in any paper deriving from this work and as such indexed in Pubmed by the journal.
Fill your name, email, and ORCID if you wish to be listed as a collaborator.

Kindly hit next if you are willing to participate.

We appreciate your participation and valuable comments.

SURG-SAT-19 Study group

Contact us:
SURG.SAT.19@Gmail.com

Effect of COVID19 Pandemic on Surgeons' Job Satisfaction and Burnout

Basic Information

1. In what country do you work?

2. What is your age? (in years)

3. What is your gender?

- Female
- Male
- Prefer not to mention

4. Which of the following best describes your current relationship status?

- Married
- Widowed
- Divorced
- Single

5. Do you have any children?

- Yes, all 18 or over
- Yes, one or more under 18
- No

6. How many years do you have in surgical practice?

7. What is your current designation?

- Resident/ Trainee
- Specialist/ Senior Specialist
- Consultant

8. What is your specialty?

- General Surgery
- Neuro Surgery
- Obstetrics and Gynecology
- Plastic Surgery
- Vascular Surgery
- Oral and Maxillofacial
- Cardiothoracic Surgery
- Urology Surgery
- Otolaryngology
- Pediatric surgery
- Orthopedics Surgery
- Other (please specify)

9. In what type of community do you work in?

- City or urban community
- Rural community

10. In what type of institution do you work?

- Academic institution
- Non-academic
- Private
- Mixed

11. If you wish to list your name in our (collaborative group) in all papers derived from this work, fill your name and Email.
(OPTIONAL and individual data will remain confidential)

Name

Email

ORCID (If Available)

Effect of COVID19 Pandemic on Surgeons' Job Satisfaction and Burnout

Job satisfaction and burn out

The aim of this part is to measure your job satisfaction before and after the pandemic. kindly give a response to each question.
in the last two questions, you can select multiple responses.

* 12. Surgery Job satisfaction before and during the pandemic

	Before the COVID-19 Pandemic	During the COVID-19 Pandemic
I will choose surgery as a career again (If I have the choice)		
I can cope with technical challenges		
I have enough case variety		
I use a full range of surgical skills in practice		
I receive adequate financial compensation		
I recommend surgery to the medical student		
Malpractice experiences have affected job satisfaction		
I feel well-respected in my community		
I can keep the right work-life balance		
I have enough time for family		
I have no issues with childcare responsibilities. (if you have children)		

* 13. Manifestations of burnout

	Before the COVID-19 Pandemic	During the COVID-19 Pandemic
I feel tired		
I have no energy for going to work in the morning		
I feel fed up		
I feel like my “batteries” are “dead”		
I feel burned out		
I feel Difficulty concentrating		
I feel I am unable to be sensitive to the needs of coworkers and patients		

Effect of COVID19 Pandemic on Surgeons' Job Satisfaction and Burnout

Changes in Career practice and resources

The following questions are related to your career practice, you are expected to give two answers on each question before and during the COVID-19 pandemic. Kindly pick the range that represents your answer.

* 14. Career practice before and during pandemic (kindly provide the approximate range)

	Before COVID 19 Pandemic	During COVID 19 Pandemic
Participation in research work (PER YEAR)		
Hours spent reading scientific articles/week		
Clinical cases in outpatient clinic/week		
Operative cases/ week		
Cases working as primary surgeon/week		
Case working as assistant surgeon/week		
Hours working on call/week		
Hours working at home/week		
Number of emergency case/week		

* 15. Role During the pandemic

	Yes	No	NA
My hospital was included to treat COVID-19 Cases			
I was included in COVID-19 patient management team			
I was in contact with COVID-19 positive cases			
I was asked to share in medical practice away from my surgical field			
I felt my clinical role was minimized during the pandemic			

* 16. How do you evaluate the availability of resources during the pandemic?

	Sufficient	Neutral	Insufficient
Surgical Training resources	◯	◯	◯
Surgical research resources	◯	◯	◯
Knowledge resources regarding pandemic	◯	◯	◯
Required skills regarding the pandemic	◯	◯	◯
Required protection during the pandemic	◯	◯	◯

17. In general, What would have helped a better situation for you during the pandemic?
